# Mid and long-term ecological impacts of ski run construction on alpine ecosystems

**DOI:** 10.1038/s41598-020-67341-7

**Published:** 2020-07-15

**Authors:** Csilla Hudek, Elena Barni, Silvia Stanchi, Michele D’Amico, Emanuele Pintaldi, Michele Freppaz

**Affiliations:** 1 Department of Agricultural, Forest and Food Sciences, University of Turin, Largo Braccini, 2, 10095 Grugliasco, Italy; 2 Department of Life Sciences and Systems Biology, University of Turin, Viale P.A. Mattioli, 25, 10125 Torino, Italy; 3 University of Turin, Natrisk, Largo Braccini, 2, 10095 Grugliasco, Italy; 4 School of Water, Energy and Environment, Cranfield University, College Rd, Cranfield, MK43 0AL UK

**Keywords:** Restoration ecology, Environmental impact

## Abstract

The proliferation of ski run construction is a worldwide trend. The machine-grading of slopes involved during ski run construction changes the physical, chemical and biological properties of the soil, having significant long-term ecological impact on the environment. Establishing and developing plant communities in these affected areas is crucial in rehabilitating the biotic and abiotic soil environment, while also improving slope stability and reducing the risk of natural hazards. This study evaluates changes in plant-soil properties and the long-term effects of machine-grading and subsequent restoration of ski runs so as to contribute to formulating the best practices in future ski run constructions. Study plots were established in 2000 and re-surveyed in 2017 on ski runs, which had been machine-graded and hydroseeded in the 1990s. Vegetation, root trait and soil surveys were carried out on ski run plots and compared to paired, undisturbed control sites off the ski runs. Plant cover remained unchanged on the ski-runs over time but plant richness and diversity considerably increased, reaching similar levels to undisturbed vegetation. Plant composition moved towards more semi-natural stages, showing a reduction in seeded plants with a comparable increase in the cover of colonizing native species. Root trait results were site-specific showing great variations between the mid and long-term after-effects of machine-grading and revegetation when compared to undisturbed sites. Under long-term management, the soil pH was still higher and the organic C content still lower in the ski runs than in the undisturbed sites, as the aggregate stability. The standard actions applied (machine-grading, storage and re-use of topsoil, hydroseeding of commercial seed mixtures, application of manure soon after seeding and low-intensity grazing) allowed the ecosystem to partially recover in three decades, and even if the soil has still a lower chemical and physical fertility than the undisturbed sites, the plant species composition reveals a satisfactory degree of renaturalization.

## Introduction

The ski industry provides an important source of income to mountain communities, attracting millions of visitors each year^1^ in a growing trend of ski run construction around the world^2^. The construction of ski runs and related infrastructures causes long-term changes in the environment, affecting many essential ecosystem properties^3^ and services^4–6^. The ecological consequences of, and the recovery from ski run construction, hugely depend on the methods used to construct the ski runs as well as on the land restoration work employed.

Machine grading is the most extensively used technique to create ski runs to achieve an even surface for skiers. This technique involves the removal of the entire vegetation cover and the soil surface together with its seedbank and soil biota as well as the displacement of boulders. After construction, the physical, chemical and biological properties of the soil undergo considerable changes^7,8^. Previous studies found increased soil compaction [e.g. 9, 10] along with higher concentrations of phosphorus, base cations and soil pH^7^. Barni et al.^9^ recorded decreased levels of total nitrogen, organic carbon and cation exchange capacity as well as a severe breakdown of soil aggregate stability on graded ski runs compared to undisturbed sites. These altered soil properties trigger erosion and affect the natural recolonization and development of plant communities, particularly on slopes above the timberline. Vegetation cover plays a crucial role in improving slope stability, reducing the risk of natural hazards and affecting the biotic and abiotic soil environment^7,11^, although feedback mechanisms during the succession are rarely distinguishable^12^. Roots increase the resistance of the soil by improving its mechanical and hydrological properties^13,14^. Aboveground vegetation reduces raindrop energy and runoff velocity by increasing surface roughness^15^ and when the aboveground vegetation is absent (e.g. winter dormancy or after grazing), root systems still contribute to soil reinforcement.

In restoring vegetation cover to these graded sites, using hydroseeding with commercial seed mixtures^9,16^ rather than local, site specific seed mixtures is common practice due to pricing and availability. Commercial seed mixtures however, require additional fertilization and can inhibit the establishment of native vegetation and their leaf litter can alter soil quality^7,17^. Thus, the selection of the appropriate combination of plants can speed up ecological restoration and prolong the process of stabilizing and recovering slopes^18^.

After construction, ski runs need to be carefully maintained and managed throughout the snow-free period. The chosen management techniques would further influence the time needed for, and the level of recovery of the ecosystem^9,19–21^ that during winter, could additionally be stressed by snow grooming (the process of moving, flattening, compacting or rototilling the snow with specialized equipment) and the use of artificial snow, both of which impede site recovery^22^. Snow grooming increases soil compaction, significantly reduces infiltration rate, and makes the soil and site more susceptible to erosion processes. Compacted snow can significantly affect the pedoclimate leading to poorer insulation. Lower soil temperatures place greater physiological stress on vegetation due to increased snow cover duration and, conversely, to a reduced growing season for plants. Körner ^23^ and Meijer zu Schlochtern et al. ^7^ reported that earlier melting sites show greater biodiversity, decomposition rate, humus content and microbial activity.

During the snow free period, cutting or grazing of vegetation is the most widely recommended management technique^20,21^, with the exclusion of the highest elevations where grazing could accelerate erosion and reduce the recovery rate. The level of initial disturbance, combined with restoration techniques and the chosen ski run management, can greatly influence the time needed for disturbed mountain slopes to recover and self-sustain^11,19^.

The ultimate goal of this study is to contribute to formulating the best practices in future ski run construction. The specific aim of this study is to evaluate the impact of machine-grading and the outcome of restoration on ski runs, by analyzing vegetation as well as soil changes over time. To achieve that, study plots were established and surveyed in 2000 followed by a re-surveying in 2017 on ski runs, constructed and revegetated in the 1990s. To determine whether the plant and soil dynamics were moving towards self-sustainable and semi-natural conditions, changes measured in study plots on ski runs were compared to adjacent reference sites off the ski run. Of particular interest was examining: (i) vascular plant species cover, richness, diversity and composition; (ii) root morphometry (iii) soil physicochemical properties (iv) potential natural hazards due to erosive processes.

## Materials and methods

### Study sites

**Figure 1:**
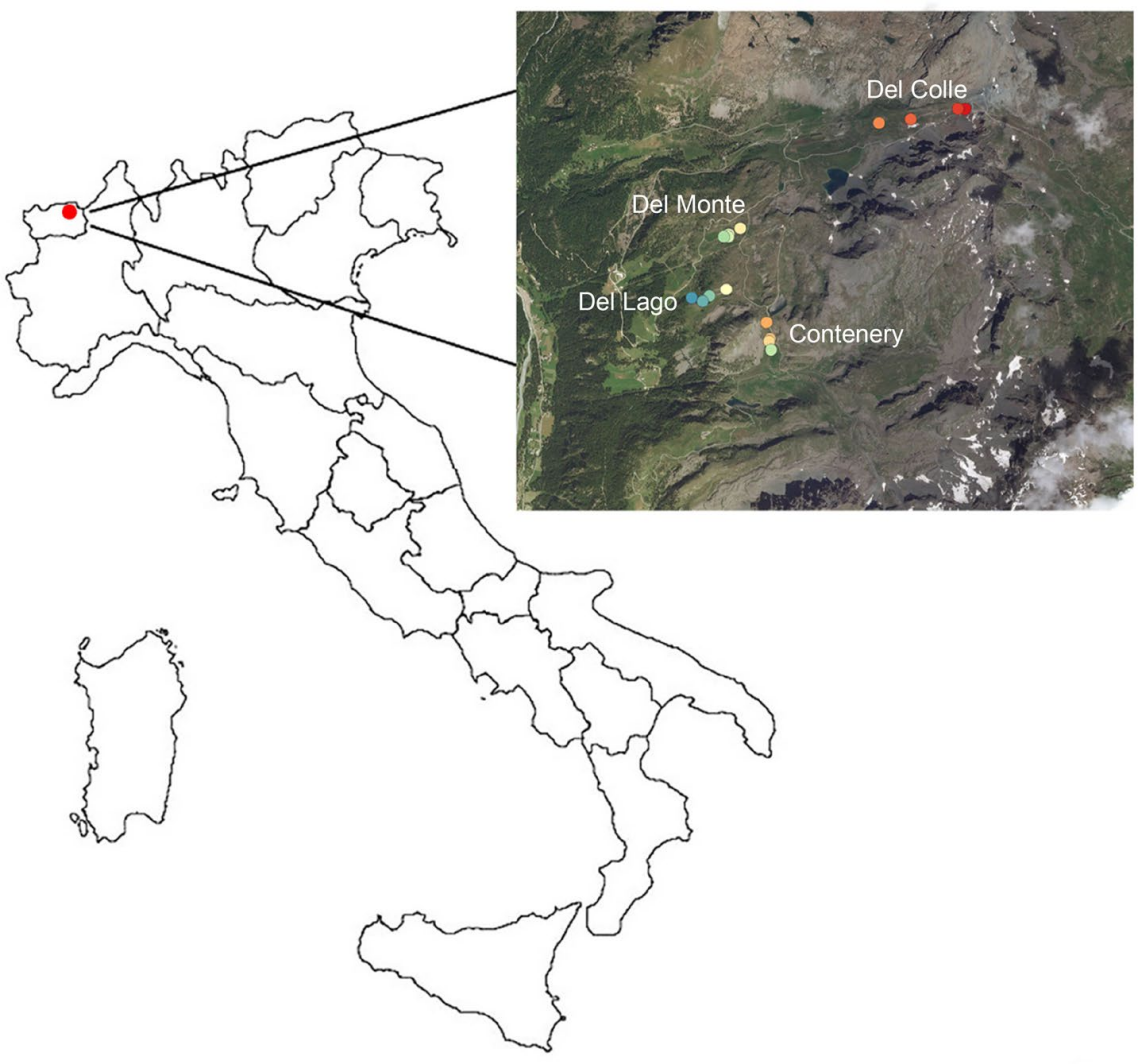
Map of the study area in the Monterosa ski resort with the plots in the ski runs; Del Colle (A, B, C, D), Contenery (E, F, G, L), Del Monte ( H, J, K, M) and Del Lago (I, N, O, P) (Aosta Valley, NW-Italian Alps). (The map was created using Q GIS software (version 3.3.0) then modified and assembled using Photoshop version (CS6)).

The research was performed on ski runs located in the Monterosa ski resort (45°51ʹ35ʺ N; 7°45ʹ35ʺ E), in the NW Italian Alps (Champoluc, Val d’Ayas, Aosta) (Fig. 1). Each ski run was paired with a control site off the ski runs, representing undisturbed conditions. The sites (ski runs and the corresponding control locations) are located between 2,100 and 2,700 m a.s.l. with an inner-alpine subcontinental climate. The average precipitation in Champoluc weather station is 722 mm year^-1^ (1,560 m a.s.l.) while the mean annual air temperature in the elevating gradient ranges between 2 and − 2 °C^24^. Maximum precipitation during May and June is 80 mm each, while the winter months are generally dry, with a mean of 130 mm (snow water equivalent) during the winter trimester^24^.

The undisturbed soils are classified as Regosols, Cambisols and Podzols according to the WRB soil classification system^25^, developed from morainic parent material composed of calcschists mixed with mafic and ultramafic rocks^9^. The natural vegetation mainly consists of acidophilus alpine grasslands and dwarf shrub heaths.

Ski runs were machine-graded between 1988 and 1996 (see Supplementary Table S1), and hydroseeded immediately after construction utilizing commercial grass/legumes mixtures^9^. Mineral fertilizer was supplied during hydroseeding (N/P/K 12/12/12, 30–40 g m^-2^) and manure was added during the second year. Snow grooming and artificial snowmaking were applied during winter and low-intensity sheep and cattle grazing during summer on all runs, yearly. No additional, active restoration work was carried out and the runs were left to regenerate without further assistance.

### Field methods

Field measurements and sampling were carried out between July and August in 2000^9^ and 2017 on four randomly chosen sampling plots along four ski runs (n = 16) and on paired control plots off the ski runs (n = 16). Detailed descriptions of the sampling plots are reported as Supplementary Table S1. The plot pairs chosen were of similar elevation, inclination and aspect; control plots were at most 50 m away from their ski run plots and had intact undisturbed soil and vegetation, since they were not affected by machine-grading and snow preparation.

### Vegetation survey

The survey was carried out on 3 × 3 m plots along the ski runs and the corresponding control sites. Top cover (%) of vascular plants and bare ground (soil + solid rock + scree) was visually estimated. Plant species rooting in the plot were identified and cover value (%) was estimated in relation to the top vascular plant cover. The observed cover value (%) of each species was directly noted and used for further calculations. Plant species richness (S), diversity (Shannon Index (H),^26^) and evenness (Shannon Evenness Index (E)) were calculated. Target species, defined as representatives of natural and semi-natural vegetation in a given region^27^ were used to gauge restoration progress in the long term. Therefore, plant species recorded in the ski run plots were divided into three functional groups: (1) seeded (*taxa* introduced in the ski runs by hydroseeding), (2) native pioneer (naturally colonizing *taxa* from siliceous scree communities), (3) native late-successional (naturally colonizing *taxa* from alpine grasslands and dwarf shrub heaths). The occurrence of a *taxon* in habitats (e.g. scree or alpine grassland) follows Flora indicativa^28^. Regional^29^ and National^30^ Red Lists were also checked for rare and threatened species. The species nomenclature complies Flora alpina^31^. The list of species and their classification into functional groups is reported as Supplementary Table S2.

Mean height of plant cover was recorded and the aboveground biomass was harvested directly above the soil surface from two randomly selected plots (15 × 15 cm), oven dried at 70 °C for 48 h and weighed.

### Root traits

Root sampling was carried out to determine rooting depth (RD), total root length (TRL), root length density (RLD), average root diameter (D), percentage of total root length across diameter classes (roots were distributed in 10 different diameter classes, 0–0.5; 0.51–1; 1.01–1.5; …4.01–4.5; > 4.51 mm) and root biomass (RBM). Three soil samples containing the root systems of the vegetation were collected using a 9 cm long and 5.6 cm diameter cylinder to harvest roots for measuring TRL, RLD and D. Soil was washed from the roots by hand under a 0.5 mm sieve and water jet. Clean roots were placed in a 15% ethanol solution and stored at 3.5 °C until further testing. The roots were scanned with a flatbed scanner and the images were analyzed with WinRhizo 2004 b software. To gain data on RBM, plots (15 × 15 cm) previously used for measuring aboveground biomass were excavated and the soil was washed from the roots by hand as mentioned above, roots were oven dried at 70 °C for 48 h and weighed on an analytical balance. A soil profile (15 × 15 cm) linked to the sampled aboveground biomass allowed the measurement and recording of RD on sites where the maximum depth of rooting was recorded.

### Soil sampling and analysis

Three soil samples were collected at each site at 0–10/15 cm soil depth (A, AC horizons in ski runs and A, AE horizons in control sites), mixed and analyzed as one composite sample. The samples were sieved at 2 mm and analyzed in the laboratory following standard methods^32^. Soil pH was determined using a 1:2.5 soil:water suspension. Total carbon and total nitrogen (TN) were measured (after grinding and sieving at 0.5 mm mesh) by dry combustion with an elemental analyzer (CE Instruments NA2100, Rodano, Italy). The carbonate content was measured by volumetric analysis of carbon dioxide liberated by a 6 M HCl solution. The Organic Carbon (TOC) was then calculated as the difference between total C measured by dry combustion and carbonate-C. Available phosphorus (P) was determined by using the Olsen extraction method, extracted with NaHCO_3_. The exchangeable magnesium (Mg), potassium (K) and calcium (Ca) were determined by flame atomic absorption spectroscopy (FAAS, Perkin Elmer 4000) after exchange with NH_4_^+^-acetate at pH 7.0. Particle size distribution was measured using the pipette method after dispersion of the samples with Na-hexametaphosphate. Barni et al.^9^ describes the detailed soil sampling methods from 2000.

### Soil aggregate stability and soil erosion

Soil aggregate stability was determined by wet sieving at different times (5, 10, 15, 20, 40 and 60 min) and fitted with the exponential model^33^:

1 $$  {\text{Y}}\left( {\text{t}} \right) = {\text{a}} + {\text{b}}\left( {1 - {\text{e}}^{{ - {\text{t}}/{\text{c}}}} } \right)  $$

where *y* is the aggregate breakdown (%), *t* is the wet sieving time (min), *b* is the aggregate loss by abrasion (%), *a* is the incipient failure of aggregates upon water saturation (%), *c* is the time–unit parameter that links the rate of breakdown to wet-sieving time. Considering that *a* values are generally negligible with respect to *b*, in coarse-textured soils, we considered the sum (*a* + *b*) as the maximum estimated aggregate loss for further analyses.

The level of soil erosion in the plots was quantified by visual observations and categorized as follows: (i) no noticeable evidence of erosion (NON), (ii) sheet erosion (Sheet) (laminar erosion processes, without rill formation) and (iii) rill erosion (Rill) (formation of rill, i.e. channels with a depth of mm or cm).

### Statistical methods

All statistical elaborations were performed using R 3.6.1 software (R Foundation for Statistical Software, Institute for Statistics and Mathematics, Vienna, Austria).

Differences in soil chemical properties, biodiversity and root traits between ski runs and control sites were evaluated using a one-way analysis of variance (ANOVA, Tukey HSD used to test differences at p < 0.05).

In order to detect differences in plant communities across elevation, land use gradients and relevant trends occurring from the time of machine-grading, vegetation types were observed using unconstrained ordination methods (NMDS,^34^, Bray–Curtis distance). The analysis were performed with metaMDS within R vegan^35^, using a Wisconsin double standardization and a maximum number of 100 runs to reach the best solution (two axis). To visualize relationships between plant communities and environmental parameters, the NMDS biplot was interpreted using a post-hoc correlation with significant soil/environmental parameters (function envfit). To assess the rate of convergence of vegetation on ski runs towards natural vegetation (i.e., shift in plant species composition from basophilous, pioneer species to climax acidophilous ones), mixed-effects models (LME4 package ^36^) were used. The considered variables were total vegetation, seeded, pioneer and late-successional species cover, bare soil and biodiversity indices, with age as a fixed effect and a nested random effect of sampling site. As there were no significant differences, nor in soil properties nor in plant cover on the control plots between 2000 and 2017, only data from 2017 were used in the statistical analysis.

## Results

### Vegetation characteristics

**Figure 2:**
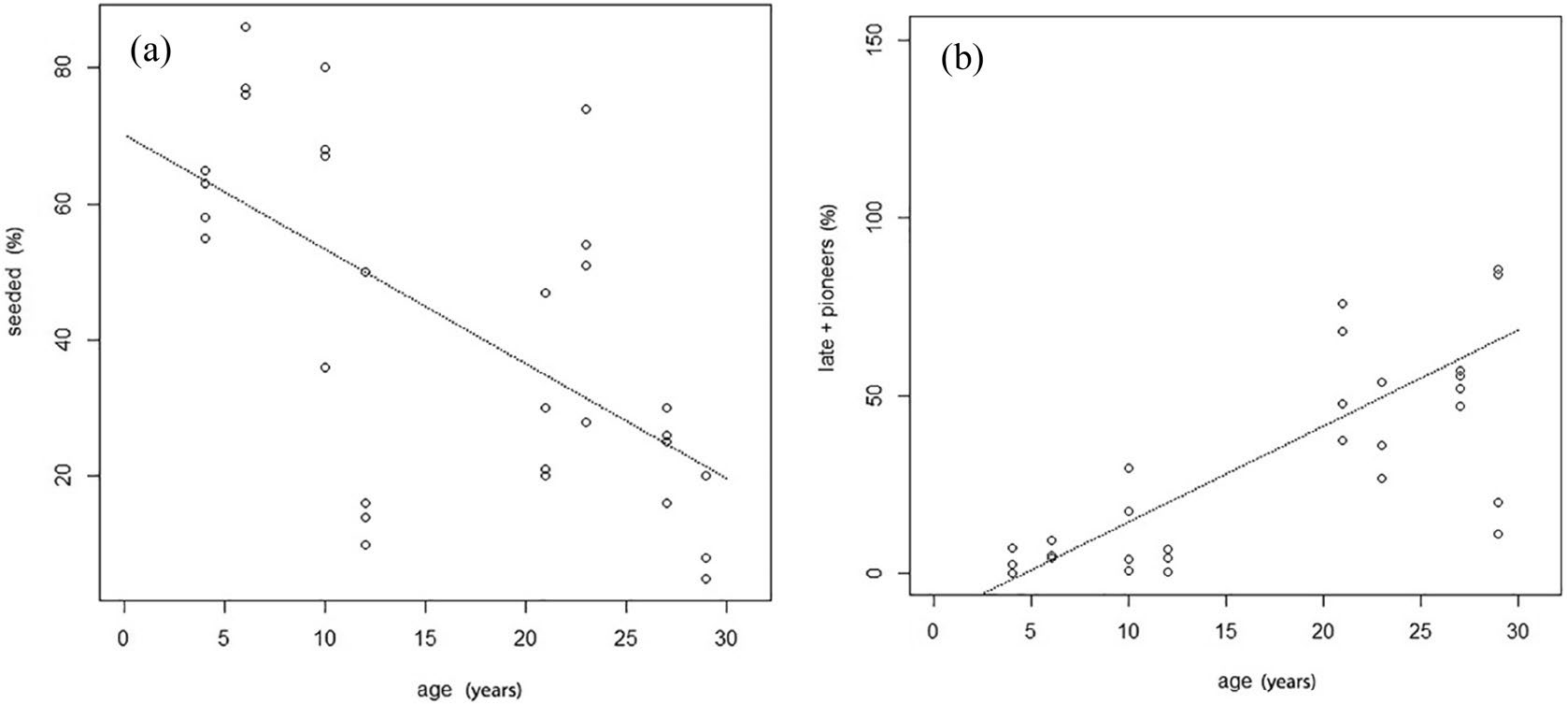
(**a)** Seeded species cover (%) and **b)** Native species cover (pioneers + late successional, %) change on ski runs correlated to years after seeding.

Total vascular plant cover did not change significantly in the ski run plots over time and maintained relatively high values (62% ± 13 in 2000 VS 72% ± 9 in 2017), not different from cover values in the undisturbed control plots (86% ± 6). Plant species richness (S_(2000)_ = 9 ± 2; S_(2017)_ = 25 ± 5; S_(control)_ = 27 ± 3), diversity (H_(2000)_ = 1; H _(2017)_ = 3; H_(control)_ = 3) as well as the evenness index (E_(2000)_ = 0,4; E_(2017)_ = 0,5; E_(control)_ = 0,6) increased considerably in the ski run plots over time (p < 0.05; see Supplementary Fig S1a, b, c), achieving values similar to those of the adjacent undisturbed communities. There was a shift in plant species composition from the first sampling (2000) to 2017. Of three plant groups (i.e. seeded, native pioneer, native late-successional species), the herbaceous layer on ski runs exhibited a clear reduction in the cover of seeded species (p < 0.001; Figure 2a), accompanied by a general recovery by native plants (p < 0.001; Figure 2b), irrespective to their ecological role (pioneer or late-successional). Increasing trends of both pioneer and late-successional species were evident (see Supplementary Fig S2a, b) but less pronounced (p < 0.05).

**Figure 3:**
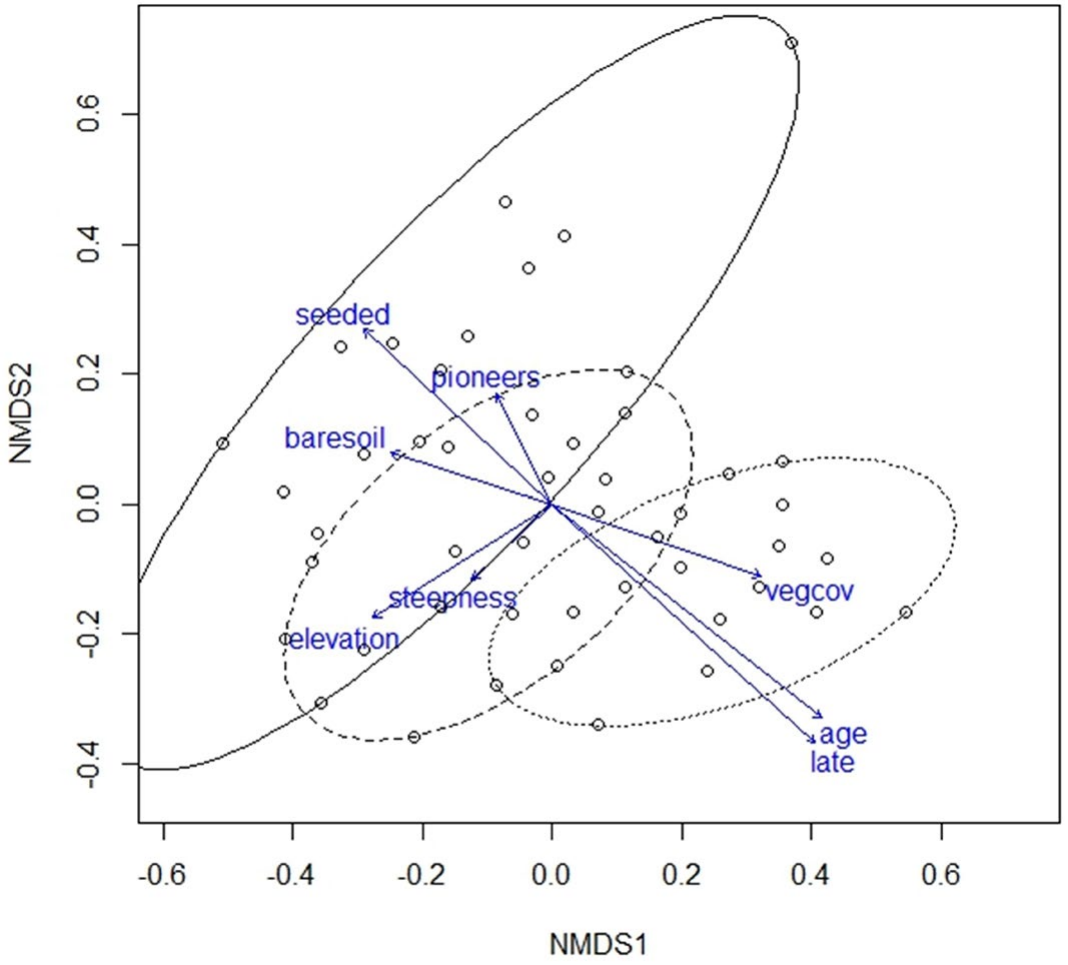
Non-metric multidimensional scaling (NMDS) of native plant (pioneer + late-successional species) cover at each study site. Ellipsis enclose samples corresponding to 2000 surveys (solid line), 2017 surveys (dashed line), control surveys (dotted line). Arrows indicate the direction of the vegetation and environmental variables explaining differences in species composition among samples.

The NMDS ordination (Fig. 3), carried out on the cover values of native plant species (i.e. pioneer + late-successional, without seeded species), showed that study sites were arranged along the first ordination axis which strongly reflected a time gradient (Table 1). On the left side of the diagram were the ski run plots surveyed in 2000, several years after machine-grading and seeding, dominated by seeded species with an uneven occurrence of pioneer species. On the right side were the undisturbed control plots, with the dominant vegetation being late-successional species. The ski run plots re-surveyed in 2017, between 20 and 30 years after ski construction and hydroseeding, appeared in the middle of the first axis, highlighting the shift in plant species composition over time.

**Table 1:** Environmental fitting significance and correlation with NMDS axis of the main environmental properties.

	NMDS1	NMDS2	R^2^	p
Elevation	− 0.36432	− 0.93127	0.2531	0.001
Steepness	− 0.25257	− 0.96758	0.0588	0.285
Age (time since machine grading)	0.99201	− 0.12615	0.5984	0.001
Seeded species cover (%)	− 0.9762	0.21689	0.3823	0.001
Pioneer species cover (%)	− 0.58247	0.81285	0.0671	0.193
Late-successional species cover (%)	0.97872	− 0.20521	0.6273	0.001
Top vegetation cover (%)	0.91634	0.4004	0.254	0.003
Bare soil	− 0.91191	− 0.41038	0.1692	0.018

The second ordination axis reflected an elevation gradient (Table 1). The earliest surveyed plots (2000) are spread broadly along this axis, showing a more uneven cover and composition of native species relating to elevation, while the latest (2017) were more gathered, similarly to control plots, indicating a vegetation cover more homogeneous with respect to elevation.

### Root properties

All ski runs had a uniformly shallow rooting depth (RD) ranging from 3.9 to 13 cm. Rooting depths were greater for control plots than for adjacent ski runs, ranging from 5 to 20 cm which in both cases significantly correlated with elevation (p < 0.05). Rooting depth results between the ski runs and control plots showed significant differences at F_7, 31_ = 4.27, p < 0.003.

The RLD results between 2000 and 2017 showed uniform decrease (ranging from 43 and 11%) on all ski runs. Higher RLD values were found on ski runs (p < 0.01) (ranging between 39.4 and 29 cm/cm^3^) when compared to the control plots (ranging between 28.6, and 14.4 cm/cm^3^) with no correlation to elevation.

**Figure 4:**
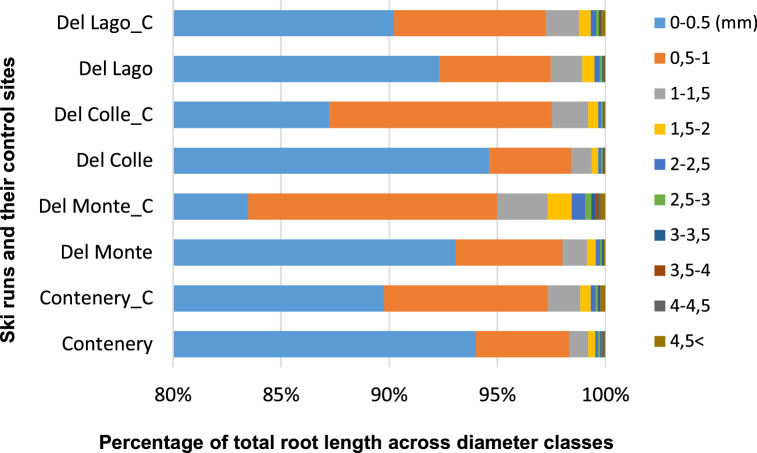
Root length distribution in different root diameter classes of the four machine-graded ski runs (Del Colle (A, B, C, D), Contenery (E, F, G, L), Del Monte ( H, J, K, M) and Del Lago (I, N, O, P)) and their corresponding undisturbed control sites (Del Colle_C (AC, BC, CC, DC), Contenery_C (EC, FC, GC, LC), Del Monte_C (HC, JC, KC, MC), Del Lago_C (IC, NC, OC, PC)) measured in 2017.

The highest mean RL (83–94%) was in the diameter class 0 < L < 0.5 (mm) for all sites followed by the class 0.51 < L < 1 (mm) with 3.7–11.4% and class 1.1 < L < 1.5 (mm) with a 2.3–0.9% (Figure 4) (year 2017). Roots in larger diameter classes 1.51 < (mm) make up less than 1% of the TRL for all ski run samples and 2% for the control plots. Results from control plots showed a higher RL distribution in higher diameter classes compared to ski run results. Apart from the highest elevation control plot, all control plots had root diameters higher than 4.51 < (mm) while results from ski runs showed that root diameter over 3.5 mm was only found on one mid-elevation ski run. The mid-term results are in line with the results from 2017. The TRL results showed significant differences between ski runs in 2000 (p < 0.01) but not in 2017 and the difference between the ski runs and their corresponding control sites were significant only at mid-altitude (2,300 m a.s.l.) (p < 0.05).

The average RBM ranged between 0.025 and 0.043 (g cm^−2^) on the ski runs and 0.055 and 0.084 (g cm^−2^) on the control sites. Even though the difference in RBM was nearly double between the control plots and the ski runs, results were not significantly higher. On all sites, RBM exceeded the aboveground biomass.

### Soil properties

The control sites were acidic (pH 3.8–5.0), with high organic carbon content (3–15%). On the undisturbed plots at the highest elevations, values of higher pH, Ca and other exchangeable bases contents and lower TOC (see Supplementary Table S3) were found, compared to other lower elevation sites, due to erosion and cryoturbation (i.e. pH values > 7.1 compared to < 5.6, and TOC < 2% compared to > 3% at lower elevations.

As in the 2000 survey, ski run samples in 2017 still showed significantly higher pH values, often exceeding 7 (p < 0.001), compared to control sites, (indicating the presence of CaCO_3_ derived from the substrate calcschists). The TOC contents in the ski runs were still significantly lower (p < 0.001), as were the TN, P Olsen and exchangeable Mg and K (p < 0.05). Exchangeable Ca and the Ca/Mg ratio in disturbed ski run soils was higher than in the undisturbed sites. The C/N ratio did not show any significant differences between sites. Soil texture was dominated everywhere by the coarsest fractions, with a clay content always below 6% (see Supplementary Table S3).

### Soil aggregate stability

As reported in the mid-term, in 2017 there was still a significantly higher (p = 0.004) maximum aggregate loss on ski runs (58%) than on control sites (34%). All soils showed comparable velocity of aggregate loss, which was relatively quick, independent on location (*c* parameter of Eq. (2), data not shown). Negative correlation was found between aggregate loss and TOC (r = -0.501, p = 0.003).

Rill erosion was dominant on most runs regardless of age and altitude and advanced sheet erosion was observed on more stable runs (see Supplementary Table S1).

## Discussion

The findings demonstrate that vegetation cover and plant diversity are negatively affected on machine-graded ski runs^10,37,38^, but in the mid-, long- term our results with the applied restoration measures showed (top-soil storage and replacement, hydroseeding and manuring instead of excessive mineral fertilization, low-intensity grazing) a successful revegetation that achieved the aim of (i) soil erosion control by a protective plant cover and (ii) a high level of colonization by native species which are more likely to self-sustain in the long term. Even though this is not supported by a negative control (ski runs without restoration) in the present study, previous restoration works on ski runs e.g.,^39,40^ showed the success of these techniques as well as other, site-specific techniques^41^ that can also be used effectively in the recovery of ski runs.

Successful site restoration for erosion control requires a 75% vegetation cover^20^, quickly establishing within the first two years and enduring in the long-term. It is unlikely to achieve good results in restoration above timberline without active species seeding^37^. Therefore, the revegetation by seed mixture cannot be omitted and to assure the best result, the use of local seed mixtures over commercial mixtures has been encouraged by experts^21,42–44^. This goal was achieved within the first years and maintained in the mid- and long-term (Table S1) in most of the study area (up to about 2,400 m), where remarkable cover values close or higher than 75% were attained.

Restored plant communities usually develops along three stages^16^: a beginning stage, in which the plant cover mainly consists of seeded species, a transitional stage, in which the autochthonous species from the surrounding begin to colonize the restored area and replace the seeded plants, and a mature stage, in which the proportion of autochthonous species is higher than that of species introduced by revegetation. In our study area, considering the consistent richness and abundance of autochthonous species (45% mean cover) compared to 26% of seeded species occurring on the ski runs, the dynamics of naturalization has reached the mature stage, after nearly thirty years.

Species introduced through commercial seed mixtures are often present on the ski runs after decades on from hydroseeding^9,11,45^, although from the initial seed mixture (13 species), only 7 were still present. This indicates the adaptive characteristics of some of the species in these seed mixtures, especially *Festuca rubra* cultivars, while others showed a very short persistence (e.g. *Festuca arundinacea* and *F. pratensis, Lolium perenne*, *Bromus inermis*). Legumes (e.g., *Trifolium pratense* and *T. repens*) still occurred in the older ski runs, with high frequencies but low cover values due to low percentages in the seed mixtures. The relative cover of seeded species decreased up to half of total vegetation cover in the long term, compared to the first years after seeding, when seeded species were still dominant. This means that autochthonous species spontaneously colonized ski-runs, improving plant richness and diversity, replacing the seeded species cover. Evidence on dynamics on re-vegetated ski runs are still scarce and contrasting: Hagen et al.^45^ found that seeded sites could inhibit the development of natural vegetation, contrary to the findings of Gretarsdottir et al.^46^.

Pioneer species can be found on ski runs (e.g. *Achillea nana* and *A. moschata* at higher or *Epilobium angustifolium* at lower elevations), showing a decreasing trend along the chronosequence of the study sites. Wipf et al.^37^ explained the abundance of pioneer species on machine-graded ski runs as a result of greater light exposure on the runs compared to off-piste sites which are afforded more shelter by vegetation, along with additional nutrients from meltwater from artificial snow. Barni et al.^9^ highlighted the high rate of seed production by some pioneer species (e.g. *Trifolium pallescens*) and the disturbance of the soil from snow grooming. Despite the sustained persistence of pioneer species, some late successional species, naturally occurring in subalpine and alpine swards (e.g. *Achillea millefolium*, *Poa alpina*, *Nardus stricta*, *Leontodon hispidus*), could establish on ski runs over the course of three decades. In fact, these species, owing to the highly efficient reproductive strategy (seed set, germination rate, vegetative propagation) are often used to enrich site-specific seed mixtures^47^. On the other hand, other dominant species found on control sites such as *Carex curvula*, a very slow growing sedge, producing heavy seeds with limited dispersal capacity and *Festuca varia* were not observed on ski runs.

These results suggest that the constitution of semi-natural plant communities can be achieved also by sowing commercial mixtures, provided that other restoration measures are applied, such as top soil conservation. Nevertheless, although the recovery appeared to perform well, the process was far from being concluded, due to the persistence of pioneer species and the absence of the species dominating in the adjacent undisturbed surroundings. The use of local seed mixtures over commercial mixtures can be successful to speed up the restoration process of the native plant community in harsher conditions^21,42–44^, but long term results are still lacking.

Soil properties results show that soil development is still in its initial stage on most parts of the studied ski runs. Higher pH values and much lower organic carbon (associated with lower exchangeable bases and nutrients) characterize ski run soils as opposed to undisturbed samples. The presence of more “primitive”, carbonate and stone-rich soils partly influences the dynamics of vegetation succession, sustaining the persistence of pioneer or early successional, base-loving species. In addition, the low P Olsen and TN level of the soil explains the abundance of pioneer species.

Alpine species generally have a shallow rooting depth due to soil temperature, water availability and stone contents^23,48^. On the studied ski runs, rooting depth was even shallower compared to the control sites. Root traits are primarily controlled by genetic characteristics^14^ but the differences in plant composition and the soil conditions^49,50^ are strongly influencing factors, creating the observed differences in root properties between ski runs and control sites. Low TOC and nutrient content is strongly associated with shallower rooting depths in reshaped soils. Soil compaction itself is a limiting factor in root growth^51^ along with decreased water holding capacity, restricting root growth into deeper layers. Machine-grading alters the entire soil structure and results in topsoil redistribution; when combined with the use of artificial snow and snow grooming, soils become increasingly compacted and lose their moisture holding capacity^10^. In order to overcome environmental stresses, roots are able to modify their properties^52,53^, which can result in shallower rooting systems.

Most roots were very fine or fine roots (< 0.5–1.5 mm in diameter) on all sites during the 2000 and 2017 surveys, which is typical of most alpine species^10,23,54^. Root length density was lower on the control sites compared to ski runs and a lower RLD was measured in lower diameter classes and higher in higher diameter classes for control sites, as the soil conditions on the ski runs inhibit the growth of species with elaborate taproot morphology. Fine, fibrous roots in general result in increased RLD and as root diameter increases RLD decreases^10,55^. Increasing species richness can be an additional factor for increased RLD^10,55^, which could be a further reason for a higher RLD in low-elevation ski runs. Fine and very fine roots are responsible for water and nutrient uptake from the soil^56^ thus soil conditions such as available water or nutrients has a great influence on root biomass. Soils on ski runs have a lower organic carbon content (associated with lower nutrients) compared to undisturbed sites, so plants adaptation to these soil conditions results in the development of a higher number of fine and very fine roots to obtain the necessary nutrients. Nagelmüller et al.^49^ reported that lower soil temperature also limits root growth but did not affect aboveground biomass that reflects on the root to shoot ratio. Snow grooming on the ski runs compacts the snow, resulting in a lower soil temperature that impacts root growth^57^ which should be taken into consideration in the selection of species used in the restoration process.

Soil aggregate stability is an important indicator of the degree of disturbance of ski runs with improved soil stability being a fundamental aim of ski run management. On ski runs where the grass species proliferate, aggregate stability is increased due to the dense, fine fibrous root system of the plants as they have a superior modifying effect on soil hydrology and soil microbial activity (increase adhesion, kinetic restructuring and filamentous binding)^58^ which ultimately enhances and increases the resistance of the soil to disruptive forces^53,59^.

Restoration techniques have a fundamental role in the rehabilitation of damaged ecosystems during ski runs construction^60^. The use of appropriate restoration techniques, such as the storage and re-use of the topsoil, selection of appropriate plant material for hydroseeding and manuring after sowing, can lead to the establishment of a sufficiently dense plant cover in the ski runs after several years^41,46,60^. Otherwise, the soil physical and chemical properties, although suitable for plant colonization, would not reach the values of the adjacent undisturbed sites^60^. The soil recovery processes are slow, due to harsh environmental and climatic conditions, but the soil environment still has favorable characteristics for plant colonization. Our findings support the importance of an integrated planning activity during ski run construction, underlining the strong interaction among plant and soil properties, especially at high elevation, suggesting the importance of a cross-sectoral planning approach including expertise in pedology and plant ecology in order to improve the effectiveness of restoration efforts.

Construction and its related infrastructural demands, especially at high elevation, can degenerate the soil ecosystem to the early stages of primary succession (parent materials outcrop) if pre-existing soil horizons are not effectively managed^21^. An important factor in avoiding excessive depletion of the ecosystem and securing the success of the restoration is the duration of the construction period coupled with appropriate topsoil management. Topsoil and vegetation cover (if present) have been shown to be vital though often neglected resources in preservation and restoration and where possible, should be carefully removed and stocked in a gentle slope/flat area covered with a geotextile^61^.

Our results indicate that, even with the applied restoration techniques, the recovery of the damaged ecosystem is rather slow; in light of this efforts should be made to lower ski run construction impacts. According to the literature^41,61^, the re-use of topsoil represents a priority and it is a precondition for native plant species to be able to recolonize the areas. In addition, most of the organic matter and nutrients are located in the topsoil^41^ which contains microorganism such as nitrogen-fixing bacteria (Rhizobium) and mycorrhiza fungi^62^, which are able, due to root symbiosis, to improve the supply of nutrients for plants, reducing therefore the need of fertilization^41^. The microorganisms also increase the biological functions of the soil^63^ and a rooting effect for better soil aggregation, thus increasing protection against erosion^64,65^. Topsoil contains all generative and vegetative propagules which make resettlement possible from the original site^41^ that could ensure a faster restoration enriched with indigenous plants, as matching or similar seed bank can hardly be accessible on the market or has high cost^66,67^.

## Conclusion

This is the first comparative research to this temporal extent into the practicalities and results of mid and long-term impacts of ski run construction through machine grading and standard, widely adopted restoration techniques. Vegetation establishment on ski runs showed a steady increase in surface cover and species composition between mid and long-term surveys. Pioneer and late successional species were found on most ski runs after thirty years although at the highest elevation vegetation establishment still remains challenging. Root properties reflect the soil conditions, soil development at the highest elevation combined with increased erosive forces on steep slopes is a slow process. Even in such conditions, the soil can be colonized by local plant species which coexists with the seeded ones. Evaluating the rate and nature of any recovery observed provides extra insight to those working in preparing and managing these areas, though it has been found that bringing about significant changes in root and soil properties can take several decades. The potential benefits in efficient construction, restoration and maintenance of ski runs are not only for commercial purposes but also in potentially reducing natural hazards and protecting the future of the surrounding ecosystem; to take responsibility for and positively shape the legacy these ski runs leave for future generations.

## Supplementary information

Supplementary file1

Supplementary file2

Supplementary file3

Supplementary file4

Supplementary file5

## Data Availability

Data will be available via Dryad Digital Repository.
