# Supplementary material for: Mid and long-term ecological impacts of ski run construction on alpine ecosystems
Source: Sci Rep. 2020 Jul 15;10:11654. doi: 10.1038/s41598-020-67341-7 (PMC7363814; doi:10.1038/s41598-020-67341-7)
Supplement: Supplementary file 1 — Supplementary file1 [file 41598_2020_67341_MOESM1_ESM.pdf]

Mid and long-term ecological impacts of ski run construction on alpine ecosystems

Csilla Hudek[a,d]*, Elena Barni[b], Silvia Stanchi[a,c], Michele D'Amico[a], Emanuele Pintaldi[a], Michele Freppaz[a,c]

[a]University of Turin, Department of Agricultural, Forest and Food Sciences, Largo Braccini, 2, 10095 Grugliasco, Italy

[b]University of Turin, Department of Life Sciences and Systems Biology, Viale P.A. Mattioli, 25, 10125 Torino, Italy

[c]University of Turin, Natrisk, Largo Braccini, 2, 10095 Grugliasco, Italy

[d]Cranfield University, School of Water, Energy and Environment, College Rd, Cranfield, MK43 0AL, United Kingdom

*Corresponding author: Csilla.Hudek@cranfield.ac.uk

Figure-1S species richness (a) Shannon index (b) and evenness (c) of native species (pioneers + late successional) and changes on ski runs correlated to years after seeding

Figure-2S pioneer species (a) and late-successional species (b) cover changes on ski runs correlated to years after seeding.

Table 1S Site characteristics of ski run and control plots: coordinates (WGS 84), elevation (m a.s.l.), aspect and steepness (°), year of construction and hydro-seeding, year of sampling. Vegetation characteristics of ski run and control plots: top cover of vascular plants (%), sum cover (%) of seeded species, sum cover (%) of pioneer species, sum cover (%) of late-successional species, top cover of bare ground (%), species richness, Shannon Index, visible erosion, Root biomass (g/cm2), Root to shoot ratio, Rooting depth (cm), Total length of roots (cm), Root length density (cm/cm3).

Table 2S List of plant taxa surveyed in the ski run plots in 2000 and 2017 and in the control plots. Taxa are grouped in three categories: introduced seeded, native pioneer and native late-successional species. Frequency (%) is the ratio between the number of plots a species is present and the total number of plots of each survey (n=16). Mean abundance is the mean cover (%) of a species among plots of the same survey. Taxa occurring with cover lower than 0,1 are denoted by +. Taxa are listed in decreasing order of frequency.

Table 3S Main physical and chemical topsoil properties of the four machine-graded ski runs and their corresponding control sites. Analyzed samples from the 2017 survey. Agg.loss is the maximum estimated aggregate loss according to equation (2), i.e. (a+b)
