# Supplementary material for: Mid and long-term ecological impacts of ski run construction on alpine ecosystems
Source: Sci Rep. 2020 Jul 15;10:11654. doi: 10.1038/s41598-020-67341-7 (PMC7363814; doi:10.1038/s41598-020-67341-7)
Supplement: Supplementary file 2 — Supplementary file2 [file 41598_2020_67341_MOESM2_ESM.pdf]

Mid and long-term ecological impacts of ski run construction on alpine ecosystems

Csilla Hudek[a,d]*, Elena Barni[b], Silvia Stanchi[a,c], Michele D'Amico[a], Emanuele Pintaldi[a], Michele Freppaz[a,c]

[a]University of Turin, Department of Agricultural, Forest and Food Sciences, Largo Braccini, 2, 10095 Grugliasco, Italy

[b]University of Turin, Department of Life Sciences and Systems Biology, Viale P.A. Mattioli, 25, 10125 Torino, Italy

[c]University of Turin, Natrisk, Largo Braccini, 2, 10095 Grugliasco, Italy

[d]Cranfield University, School of Water, Energy and Environment, College Rd, Cranfield, MK43 0AL, United Kingdom

*Corresponding author: Csilla.Hudek@cranfield.ac.uk

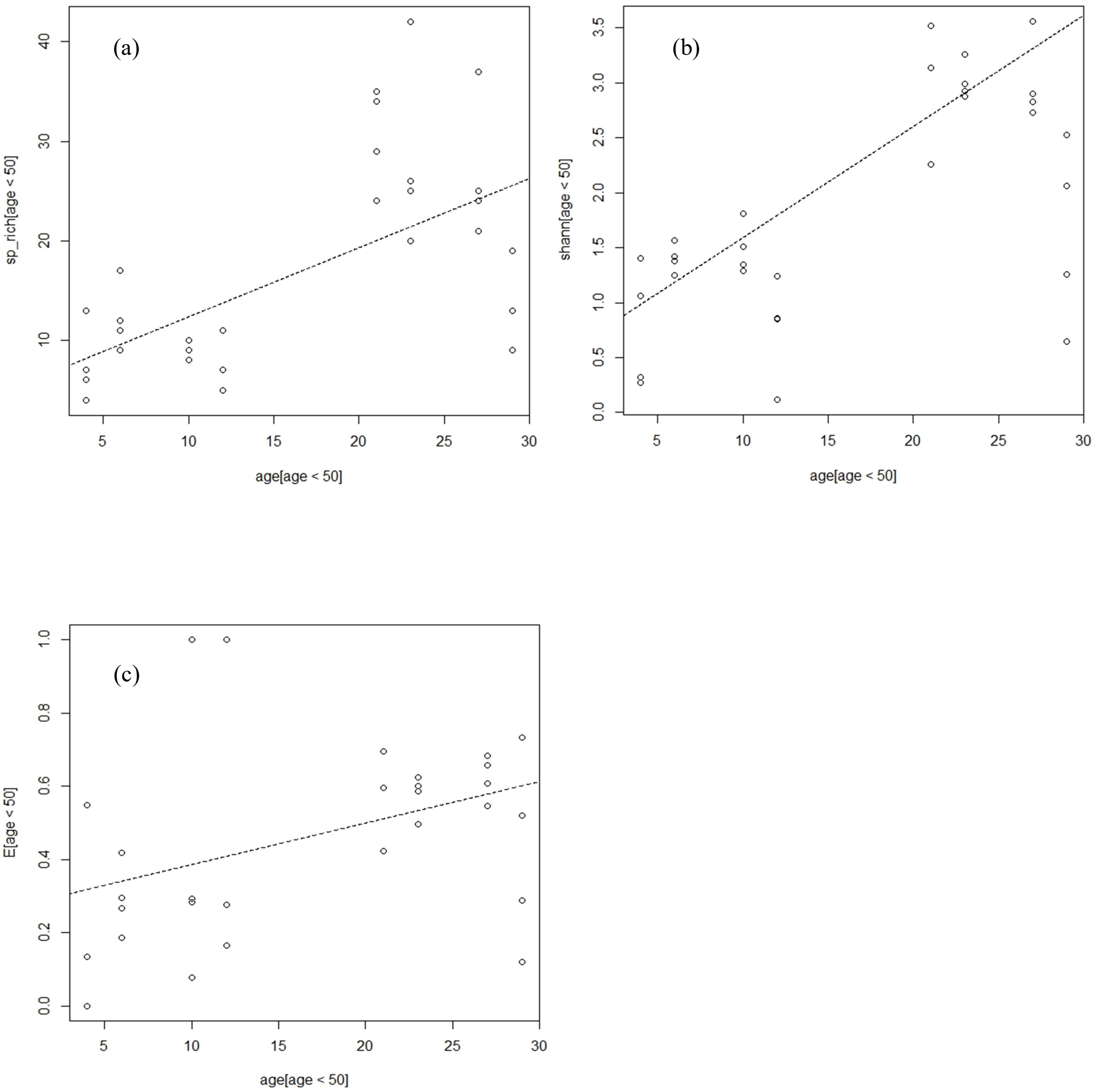

Figure-1S species richness (a) Shannon index (b) and evenness (c) of native species (pioneers + late successional) and changes on ski runs correlated to years after seeding.

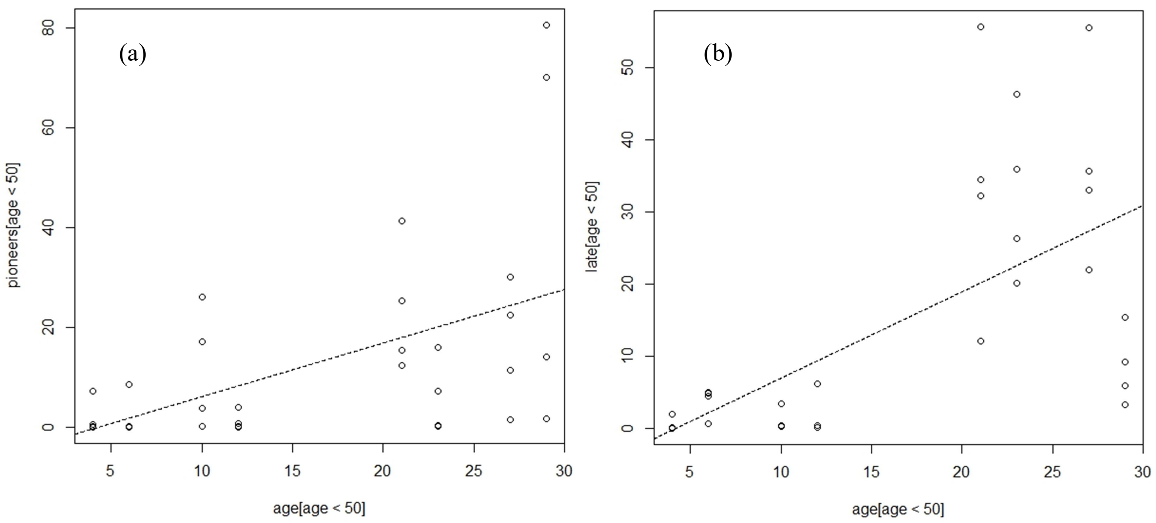

Figure- S2 pioneer species (a) and late-successional species (b) cover changes on ski runs correlated to years after seeding.
